# Initial Estimate Selection Method in Passive TDOA-Based Iterative Position Estimation Algorithms

**DOI:** 10.3390/s26144431

**Published:** 2026-07-12

**Authors:** Barbara Kaczmarek, Bartłomiej Główczyk, Mariusz Zieja

**Affiliations:** 1 IT Logistics Support Division, Air Force Institute of Technology, 01-494 Warsaw, Poland; bartlomiej.glowczyk@itwl.pl; 2 Faculty of Mechatronics, Armament and Aerospace, Military University of Technology, 00-908 Warsaw, Poland; mariusz.zieja@wat.edu.pl

**Keywords:** passive localization, position estimation, Newton–Raphson algorithm, TDOA, initial guess

## Abstract

Iterative position estimation algorithms based on Time Difference of Arrival (TDOA) are widely used in passive localization systems, including underwater acoustic networks and wireless sensor networks. A critical but often overlooked factor in their practical deployment is the selection of the initial estimate, which directly determines whether the iterative algorithm converges to the correct solution. This paper presents a case-specific approach to initial estimate selection in passive TDOA-based iterative position estimation algorithms. The study evaluates two proposed methods against a common baseline approach, where the initial guess is placed at the center of the sensor formation. Simulations were conducted in Python for both 2D and 3D scenarios, with sensors arranged in two different geometric configurations. A grid-based analysis over a 2 × 2 km area was used to assess performance under both noise-free and noisy TDOA conditions, with Gaussian-distributed error introduced at varying standard deviations. The results demonstrate that in regions where convergence is sensitive to initialization, the proposed Method 1 significantly improves reliability, especially for asymmetric sensor configurations. These findings highlight the importance of initial estimate selection to enhance position estimation accuracy and robustness, particularly in passive systems with limited prior information.

## 1. Introduction

Passive position estimation is a growing area of research, no longer limited to military applications. Its key advantage lies in the absence of an emitted signal that could be detected and tracked by an enemy [1]. Increasingly, passive position estimation is also being used in civilian applications, utilizing ambient radio frequency (RF) signals such as Wi-Fi [2,3,4], LTE [5,6,7], and acoustic signals generated by the localized object itself, including underwater vehicles operating in swarms [8].

In this paper, a Time Difference of Arrival (TDOA)-based position estimation technique [9] is analyzed in combination with iterative nonlinear equation-solving methods. Specifically, the Newton–Raphson (NR) iterative method is chosen for evaluation purposes.

In general, iterative algorithms provide approximate solutions that are improved with each iteration until a specified error threshold is met. A crucial factor influencing the convergence of these algorithms is the selection of the initial estimate (commonly called the initial guess), which must be sufficiently close to the actual solution. The most common practical approach is to use the geometric center of the sensor formation as this initial estimate. While intuitive, this choice does not guarantee convergence across the entire search area—regions of NR divergence occur at specific locations depending on sensor geometry and the structure of TDOA sign change boundaries [10,11] and cannot be predicted without prior knowledge of the target location, which is unavailable in passive localization. Asymmetric sensor configurations are particularly affected, as demonstrated in the simulation results of this paper. When the target position is unknown—as in position estimation problems—choosing a suitable starting point therefore becomes particularly challenging.

Several mathematical approaches for initial guess selection have been proposed in the literature (e.g., Refs. [12,13]). However, those solutions often assume ideal conditions, such as uninterrupted, noise-free data, which rarely hold in real-world scenarios. In reality, position estimation systems must cope with measurement noise, data gaps, and signal interference, all of which can degrade accuracy and complicate algorithm design.

On the other hand, some case-specific solutions related to convergence in other domains have also been discussed in the literature, some of which are mentioned in Ref. [12] and are related to convergence of the solution of visco-plastic models [14], magnetostatic problems [15], and electrical power flow problems [16]. In Ref. [17], an interesting method for selecting starting points for iterative position estimation algorithms using feedforward neural networks is presented, along with a thorough analysis of related work. The authors conclude that many articles address position estimation using neural networks, while none specifically focus on starting point selection.

In this paper, a new case-specific approach is proposed in connection to initial estimate selection in passive TDOA-based iterative position estimation algorithms, particularly for cases where sensors are located at approximately the same height.

**Figure 1:**
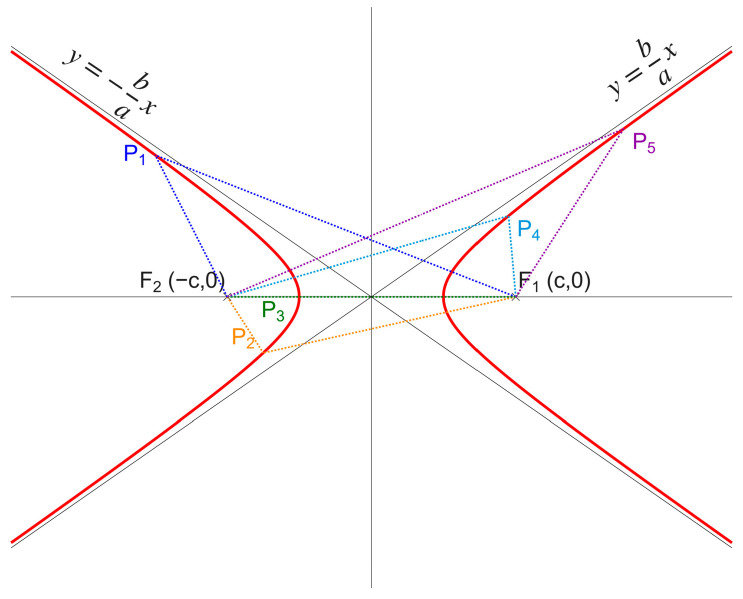
Hyperbola with foci in point F1 and F2.

**Figure 2:**
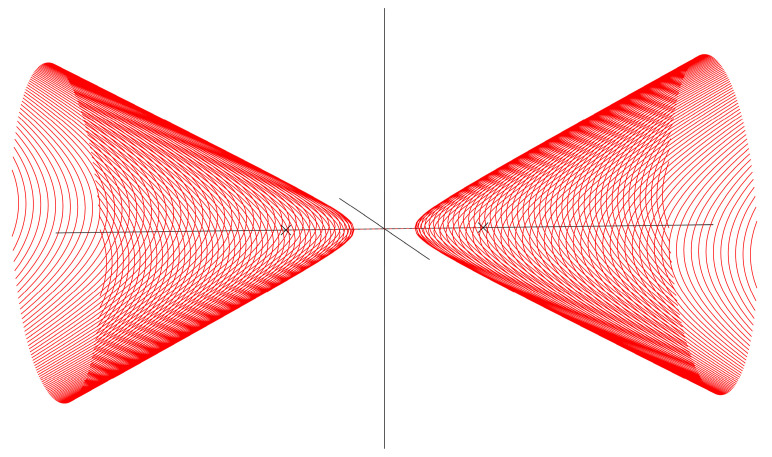
Circular hyperboloid of two sheets.

**Figure 3:**
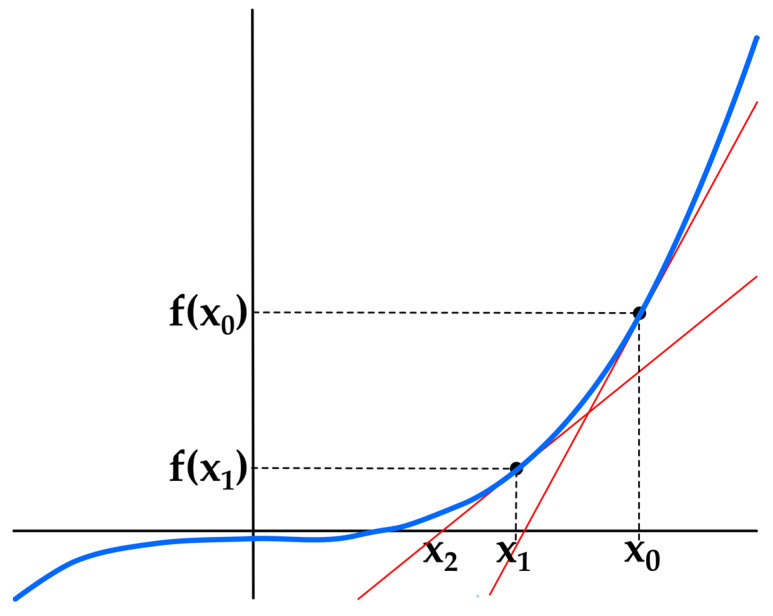
NR method with one variable geometrical representation.

**Figure 4:**
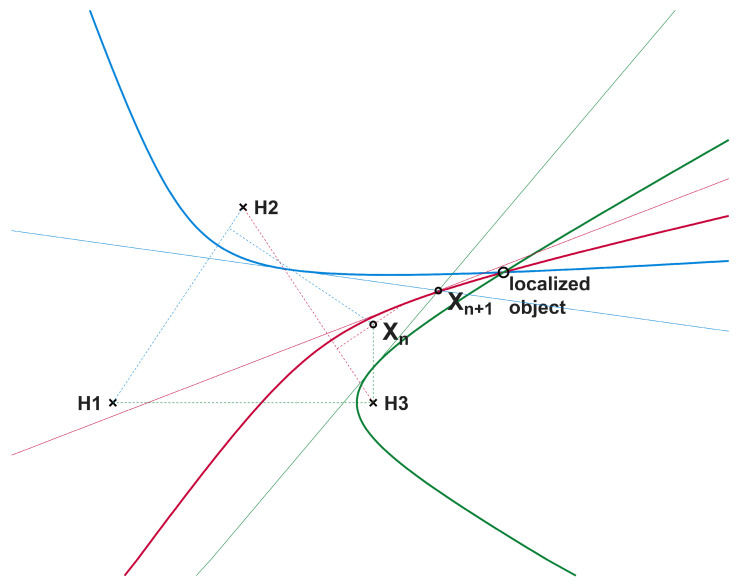
NR method for geometrical representation for three sensors and 2D scenario.

**Figure 5:**
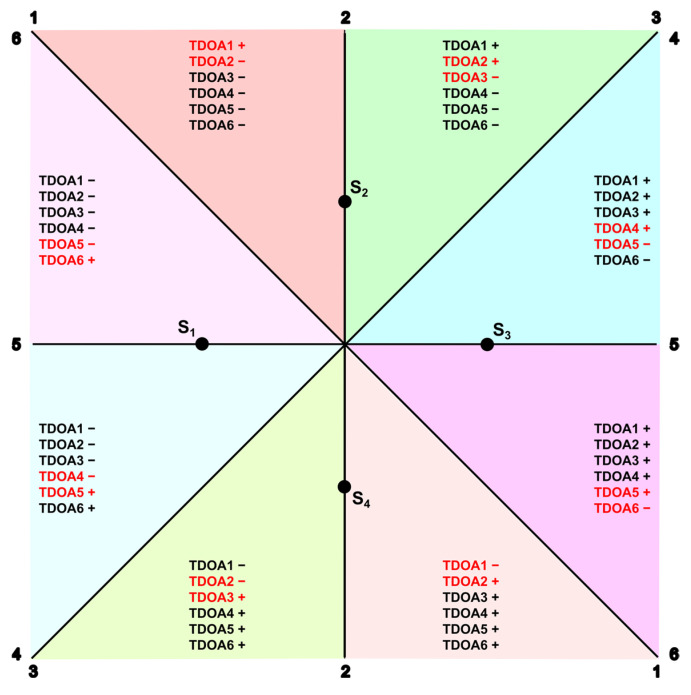
Narrowed areas for square geometry.

**Figure 6:**
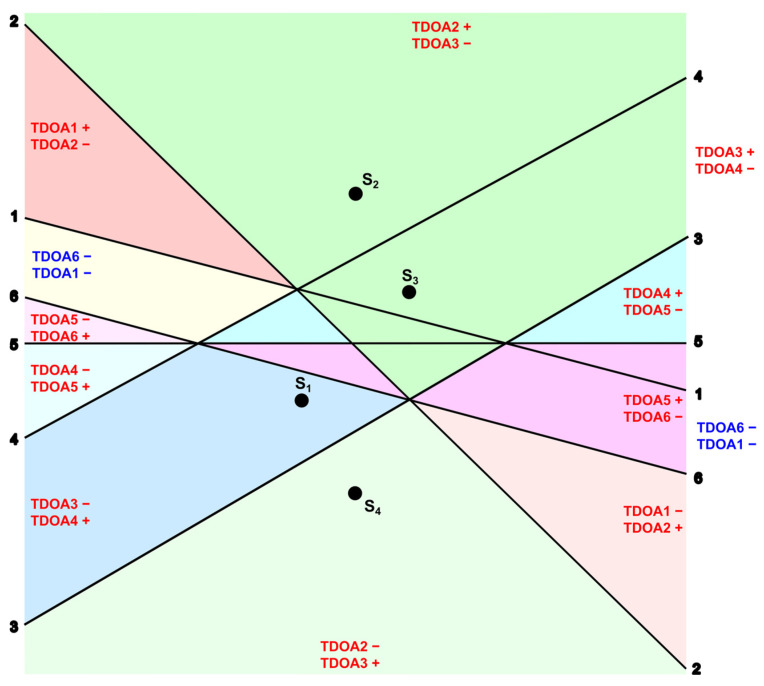
Narrowed areas for parallelogram geometry.

**Figure 7:**
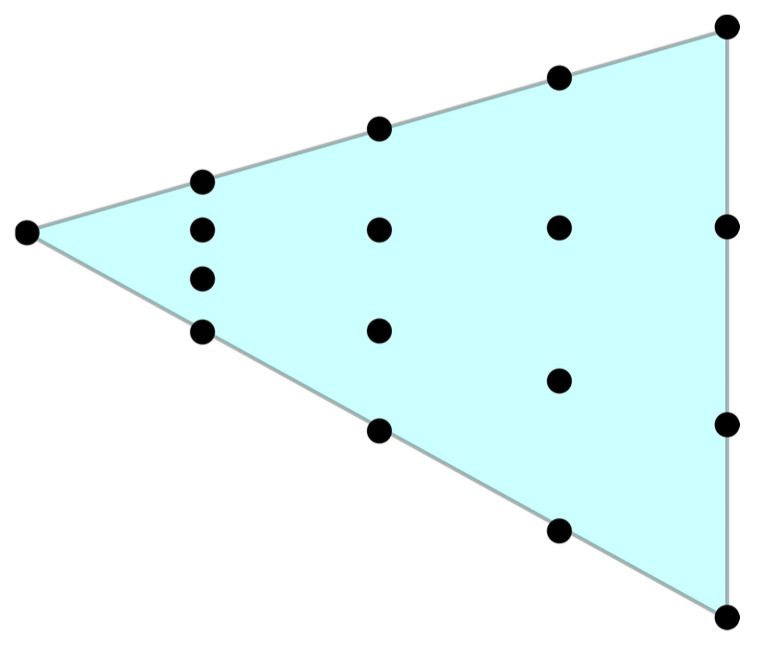
Initial Guesses location in the selected area.

Figures presented in the theoretical sections of this paper were prepared using MATLAB Online (basic) for Figures 1, 2 and 4, and Inkscape (v1.3.2) for Figures 3, 5, 6 and 7. Minor graphical corrections to remaining figures were also applied using Inkscape (v1.3.2).

## 2. TDOA Basis

The TDOA method, also known as multilateration or the hyperbolic system, is a position estimation technique based on measuring the time difference of signal arrival between two receivers.

This system exploits the fact that the difference in distance from any point on a hyperbola to its two fixed points, which form its foci, remains constant.

(1) $$\left|\bar{{F_{1}P}_{n}}-\bar{{F_{2}P}_{n}}\right|=const=TDOA$$

where TDOA denotes the time difference of arrival expressed as a distance (c·Δt) in meters, with c being the signal propagation speed.

The hyperbola shown in Figure 1 is described by the following equation:

(2) $$\frac{x^{2}}{a^{2}}-\frac{y^{2}}{b^{2}}=1$$

where for multilateration purposes:

(3) $$a=\frac{1}{2}TDOA$$

(4) $$c=\frac{1}{2}\bar{F_{1}F_{2}}$$

(5) $$b^{2}=c^{2}-a^{2}$$

To address the 3D solution, the system relies on the fact that the difference in distance from any point on a circular hyperboloid of two sheets to its two fixed points, which form its foci, remains constant.

The general equation of a hyperboloid of two sheets has the following form [18]:

(6) $$\frac{x^{2}}{A^{2}}-\frac{y^{2}}{B^{2}}-\frac{z^{2}}{C^{2}}=1$$

On the other hand, by rotating the hyperbola around its real axis, we obtain a surface, called a two-shell rotating circular hyperboloid (Figure 2), which can be described by the formula:

(7) $$\frac{x^{2}}{a^{2}}-\frac{y^{2}}{b^{2}}-\frac{z^{2}}{b^{2}}=1$$

where a, b, and c are described by Formulas (3)–(5).

It should also be noted that the process of determining TDOA values is most commonly carried out using the classical cross-correlation function (CCF) [19]. Many papers present alternative methods for determining TDOA, either in the form of new techniques or, more commonly, as modifications of the cross-correlation function, such as those described in Refs. [20,21,22].

## 3. Newton–Raphson Method

The Newton–Raphson (NR) method is an iterative method for finding the roots of a function, i.e., for obtaining a solution to the equation $f\left(x\right)=0$. The history of the method development can be found, e.g., in Ref. [23]. Over the years, the method has become a very important tool applied to solve the problems in different areas, including statistics, economics, finance, and engineering, among others [24]. Moreover, the NR method has undergone a number of modifications to improve its capabilities, e.g., like those described in Ref. [25].

For one variable, the idea of the NR method begins with an initial estimate $x_{0}$ for a root of given function $f\left(x\right)$. The further approximations are calculated according to:

(8) $$x_{n+1}=x_{n}-\frac{f\left(x_{n}\right)}{f^{\prime }\left(x_{n}\right)}$$

The convergence process for one variable is shown in Figure 3.

For 2D position estimation purposes with N sensors, multilateration function has the format shown in Ref. (9), and the process of finding a solution in the following iteration can be shown in geometrical representation, as presented in Figure 4. Each color represents one TDOA measurement pair and its corresponding geometric elements.

(9) $$f_{k}\left(x,y\right)=\sqrt{{\left(x-x_{H_{i}}\right)}^{2}+{\left(y-y_{H_{i}}\right)}^{2}}-\sqrt{{\left(x-x_{H_{j}}\right)}^{2}+{\left(y-y_{H_{j}}\right)}^{2}}-{TDOA}_{ij}=0$$

where i=1,2,…,N−1; j=2,3,…,N; k=1, 2,…,m; $m=\;\frac{N\left(N-1\right)}{2}$

For a 2D scenario, the estimated result, function, and Jacobian matrix are defined as:

(10) $$x=\left[\begin{array}{c} x\\ y \end{array}\right]$$

(11) $$F\left(x\right)=\left[\begin{array}{c} f_{1}\left(x,y\right)\\ f_{2}\left(x,y\right)\\ \vdots \\ f_{m}\left(x,y\right) \end{array}\right]$$

(12) $$J\left(x\right)=\left[\begin{array}{cc} \frac{\partial f_{1}}{\partial x} & \frac{\partial f_{1}}{\partial y}\\ \frac{\partial f_{2}}{\partial x} & \frac{\partial f_{2}}{\partial y}\\ \vdots & \vdots \\ \frac{\partial f_{m}}{\partial x} & \frac{\partial f_{m}}{\partial y} \end{array}\right]$$

where:

(13) $$\frac{\partial f_{k}}{\partial x}=\frac{x-x_{H_{i}}}{\sqrt{{\left(x-x_{H_{i}}\right)}^{2}+{\left(y-y_{H_{i}}\right)}^{2}}}-\frac{x-x_{H_{j}}}{\sqrt{{\left(x-x_{H_{j}}\right)}^{2}+{\left(y-y_{H_{j}}\right)}^{2}}}$$

(14) $$\frac{\partial f_{k}}{\partial y}=\frac{y-y_{H_{i}}}{\sqrt{{\left(x-x_{H_{i}}\right)}^{2}+{\left(y-y_{H_{i}}\right)}^{2}}}-\frac{y-y_{H_{j}}}{\sqrt{{\left(x-x_{H_{j}}\right)}^{2}+{\left(y-y_{H_{j}}\right)}^{2}}}$$

Finally, the further NR approximations are calculated in accordance with:

(15) $$x_{n+1}=x_{n}-{J\left(x_{n}\right)}^{-1}F\left(x_{n}\right)$$

In the general case where the system is overdetermined (i.e., the number of TDOA equations m exceeds the number of unknowns), the Jacobian J is not square and ${J^{+}=\left(J^{T}J\right)}^{-1}J^{T}$ is used instead of $J^{-1}$.

The complete Newton-Raphson procedure is summarized in Algorithm 1.
**Algorithm 1.** Newton–Raphson algorithm**Input:** Initial.Guess, $sensor.coord=\left[\begin{array}{cccc} x_{H_{1}} & x_{H_{2}} & \ldots & x_{H_{N}} \end{array}\right]\;$,     $TDOA=\;\left[\begin{array}{ccccc} TDOA1 & TDOA2 & \ldots & TDOAm & TDOA1 \end{array}\right]$, error.limit, It.limitCalculate F and J value for Initial.Guess with (11) and (12)$\mathrm{Calculate}\;error=max\left(\left|F\right|\right)$Set error.diff=errorSet It=0while error≥error.limit **and** It<It.limit **and** error.diff≠0 **do**   $\;\mathrm{Calculate}\;V=-J^{-1}F$    Calculate Initial.Guess= Initial.Guess+V    Calculate F and J value for new Initial.Guess with (11) and (12)   $\;\mathrm{Calculate}\;error.diff=max\left(\left|F\right|\right)-error$   $\;\mathrm{Calculate}\;error=max\left(\left|F\right|\right)$    Set It=It+1**end while****Output:** Position=Initial.Guess

In an analogous manner, the 3D solution is calculated with function calculated as:

(16) $$f_{k}\left(x,y,z\right)=\sqrt{{\left(x-x_{H_{i}}\right)}^{2}+{\left(y-y_{H_{i}}\right)}^{2}+{\left(z-z_{H_{i}}\right)}^{2}}-\sqrt{{\left(x-x_{H_{j}}\right)}^{2}+{\left(y-y_{H_{j}}\right)}^{2}+{\left(z-z_{H_{j}}\right)}^{2}}-{TDOA}_{ij}=0$$

Furthermore, the estimated result, function, and Jacobian matrix are defined as:

(17) $$x=\left[\begin{array}{c} x\\ y\\ z \end{array}\right]$$

(18) $$F\left(x\right)=\left[\begin{array}{c} f_{1}\left(x,y,z\right)\\ f_{2}\left(x,y,z\right)\\ \vdots \\ f_{m}\left(x,y,z\right) \end{array}\right]$$

(19) $$J\left(x\right)=\left[\begin{array}{ccc} \frac{\partial f_{1}}{\partial x} & \frac{\partial f_{1}}{\partial y} & \frac{\partial f_{1}}{\partial z}\\ \frac{\partial f_{2}}{\partial x} & \frac{\partial f_{2}}{\partial y} & \frac{\partial f_{3}}{\partial z}\\ \vdots & \vdots & \vdots \\ \frac{\partial f_{m}}{\partial x} & \frac{\partial f_{m}}{\partial y} & \frac{\partial f_{m}}{\partial z} \end{array}\right]$$

where:

(20) $$\frac{\partial f_{k}}{\partial x}=\frac{x-x_{H_{i}}}{\sqrt{{\left(x-x_{H_{i}}\right)}^{2}+{\left(y-y_{H_{i}}\right)}^{2}+{\left(z-z_{H_{i}}\right)}^{2}}}-\frac{x-x_{H_{j}}}{\sqrt{{\left(x-x_{H_{j}}\right)}^{2}+{\left(y-y_{H_{j}}\right)}^{2}+{\left(z-z_{H_{j}}\right)}^{2}}}$$

(21) $$\frac{\partial f_{k}}{\partial y}=\frac{y-y_{H_{i}}}{\sqrt{{\left(x-x_{H_{i}}\right)}^{2}+{\left(y-y_{H_{i}}\right)}^{2}+{\left(z-z_{H_{i}}\right)}^{2}}}-\frac{y-y_{H_{j}}}{\sqrt{{\left(x-x_{H_{j}}\right)}^{2}+{\left(y-y_{H_{j}}\right)}^{2}+{\left(z-z_{H_{j}}\right)}^{2}}}$$

(22) $$\frac{\partial f_{k}}{\partial z}=\frac{z-z_{H_{i}}}{\sqrt{{\left(x-x_{H_{i}}\right)}^{2}+{\left(y-y_{H_{i}}\right)}^{2}+{\left(z-z_{H_{i}}\right)}^{2}}}-\frac{z-z_{H_{j}}}{\sqrt{{\left(x-x_{H_{j}}\right)}^{2}+{\left(y-y_{H_{j}}\right)}^{2}+{\left(z-z_{H_{j}}\right)}^{2}}}$$

In a 3D scenario, first, the NR algorithm for the 2D option is calculated, and then the 3D option with an initial estimate from the 2D result.

## 4. Initial Estimate Selection Method

In many practical cases, the mean position of all sensor locations is often used as the initial estimate for iterative position estimation algorithms. This approach can lead to correct solutions, especially when the target is located between the sensors. However, the goal is to develop an initial estimate selection method that is independent of sensor geometry and not limited to specific target position layout.

Another solution is to test multiple initial guesses, either evenly distributed throughout the search area or based on pre-established rules, and then choose the one that satisfies a predefined condition. However, this approach significantly increases computational time and can still deviate considerably from the true target position.

The proposed method is based on the analysis of the TDOA sign, which enables a reduction of the search area for the target location. By narrowing the search area, the risk of converging to a solution far from the actual target position is significantly reduced, assuming a sufficient number of measurements, as required by the hyperbolic method. Within the defined area, multiple initial points are distributed along its edges and interior. At the end, the final solution must be selected. Two methods for this part of the algorithm will be presented and evaluated.

### 4.1. Selection of a Narrowed Search Area

As already mentioned, the selection of a narrowed search area is based on TDOA sign analysis. The analysis begins with a 2D scenario with four sensors spaced evenly on the square geometry, which is an easy-to-interpret situation.

Assuming four sensors:

(23) $$\begin{array}{c} TDOA1={TDOA}_{12}=d_{H1}-d_{H2}\\ TDOA2={TDOA}_{13}=d_{H1}-d_{H3}\\ TDOA3={TDOA}_{14}=d_{H1}-d_{H4}\\ TDOA4={TDOA}_{23}=d_{H2}-d_{H3}\\ TDOA5={TDOA}_{24}=d_{H2}-d_{H4}\\ TDOA6={TDOA}_{34}=d_{H3}-d_{H4} \end{array}$$

where: $d_{Hi}$—distance from sensor Hi to the localized object.

The general principle for selecting a narrowed search area is illustrated in Figure 5, which shows four sensors—H1, H2, H3, and H4—positioned at the corners of a square. The straight lines, numbered along the edges, represent TDOA sign change boundaries, with corresponding sign labels. The signs of all six TDOA combinations are indicated within each area.

It can be observed that analyzing all TDOA signs is not necessary—only the two marked in red are sufficient for identifying the area. The TDOAs that require analysis in a given area are those whose sign change lines are closest to that area. Additionally, when evaluating regions in a clockwise order, it is sufficient to check the signs of two consecutive TDOAs and determine whether their signs differ.

The situation becomes slightly more complex—but still manageable—when the sensor geometry is different. In such cases, more configurations need to be analyzed, as there may be areas where the TDOAs whose sign change lines are closest to that area share the same sign. This situation is illustrated in Figure 6. For better visibility, only relevant TDOA signs are shown. While this increases the number of areas to be searched, their number remains limited and manageable.

### 4.2. Algorithm

The algorithm presented below is only one of many possible options. In this case, the following areas are chosen clockwise. However, there are many other possibilities—all TDOA sign checks could be managed in one for loop or the area could be arranged to start from the one with the smallest cross angle or the cross angle closest to the middle of the formation. However, these options are not the subject of this present study.

The initial guess selection procedure is summarized in Algorithm 2.
**Algorithm 2.** Initial Guesses matrix selection algorithm**Input:** $sensor.coord=\left[\begin{array}{cccc} x_{H_{1}} & x_{H_{2}} & \ldots & x_{H_{N}} \end{array}\right]\;$,     $TDOA=\;\left[\begin{array}{ccccc} TDOA1 & TDOA2 & \ldots & TDOAm & TDOA1 \end{array}\right]$, rangeArrange sensors and relevant TDOA clockwiseif all TDOA≠0 **do**   $\mathrm{for}\;i=1:length\left(TDOA\right)$ **do**       $\mathrm{if}\;TDOA\left(i+1\right)>0$ and $TDOA\left(i\right)<0$ **do**           Determine the set of Initial Guesses in the selected area           **exit loop**       **end if**   **end for**   **if** no guesses found **do**       $\mathrm{for}\;i=1:length\left(TDOA\right)$ **do**           $\mathrm{if}\;TDOA\left(i+1\right)<0$ and $TDOA\left(i\right)>0$ **do**                Determine the set of Initial Guesses in the selected area                **exit loop**           **end if**       **end for**   **end if**   **if** no guesses found **do**       $\mathrm{for}\;i=1:length\left(TDOA\right)$ **do**           $\mathrm{if}\;TDOA\left(i+1\right)>0$ and $TDOA\left(i\right)>0$ **do**                Determine the set of Initial Guesses in the selected area                **exit loop**           **end if**       **end for**   **end if**   **if** no guesses found **do**       $\mathrm{for}\;i=1:length\left(TDOA\right)$ **do**
           $\mathrm{if}\;TDOA\left(i+1\right)<0$ and $TDOA\left(i\right)<0$ **do**                Determine the set of Initial Guesses in the selected area                **exit loop**           **end if**       **end for**   **end if**elseif one TDOA=0 or (multiple TDOA=0 and all cross points are out of range) **do**   Determine the set of Initial Guess’s on TDOA = 0 line(s) starting from cross point**else do**    Determine one Initial point as cross point of TDOA=0 lines**end if****Output:** Initial.Guesses

For the purposes of this article, the set of Initial Guesses in the selected area is chosen, as shown in Figure 7.

### 4.3. Proposed Methods

When Initial Guesses are established, there are at least two ways to manage them:

1. Calculate the location for each Initial Guess and choose the one with the smallest $max\left(\left|F\right|\right)$ value located within the searching range;
2. From the selected Initial Guesses, choose the one with the smallest $max\left(\left|F\right|\right)$ value and calculate the location only once.

The two methods described above will be referred to as Method 1 and Method 2 in the following test results. It should be noted that Method 2 is empirical in nature, as a smaller $max\left(\left|F\right|\right)$ at the initial point does not theoretically guarantee better convergence in nonlinear TDOA systems.

## 5. Results

The simulations were prepared in Python (v. 3.13) for two scenarios: 2D and 3D. In both cases, the sensors were positioned using two sensor geometry variants along a circle with the same radii (500 m). The localized object was placed within a 2 × 2 km area, with a grid resolution of 10 m in both the x and y directions. For the 3D simulations, two different target depth levels were additionally considered. Equivalent results would apply for positive Z values. However, due to the nature of the project within which this research was conducted, the simulated Z values are negative.

For each target point, a set of  c·TDOA values [m] were calculated, and a random value drawn from a normal distribution with zero mean and varying standard deviation (σ) was added.

For each target location, basic statistical metrics (minimum, maximum value, mean, median, standard deviation, and the percentage of estimates with an error exceeding 50 m) were calculated over the entire simulated area.

In the presented figures, the vertical and horizontal axes represent the true target location, while the heat scale represents the estimated position error, calculated as follows:

(24) $${∆}_{XY}\;=\;\sqrt{{\left(x-x_{NG}\right)}^{2}+{\left(y-y_{NG}\right)}^{2}}$$

(25) $${∆}_{Z}=\left|z-z_{NG}\right|$$

where: (x,y,z)—real target position. ($x_{NG},y_{NG},z_{NG})$—target position estimated using NG algorithm.

### 5.1. Two-Dimensional Simulations

All 2D simulations were conducted using four sensors.

In each figure, the applied initial position selection methods are labeled as follows:

(a) Initial Guess in the middle of sensor formation (further named “Guess 0”);
(b) Proposed Method 1;
(c) Proposed Method 2.

**Figure 8:**
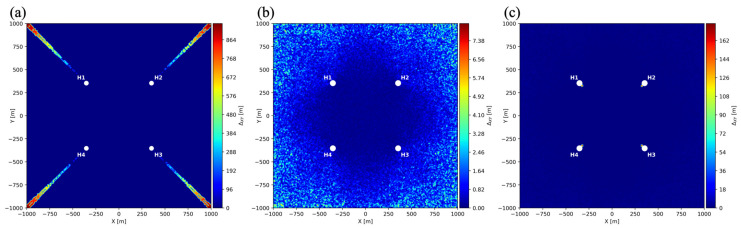
Estimated position error for TDOA values with σ=0.3 m and square sensor geometry (variant 1).

**Figure 9:**
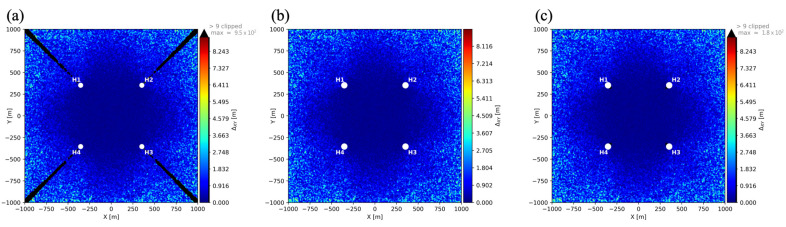
Estimated position error for TDOA values with σ=0.3 m and variant 1 sensor geometry with a max error of 9 m.

**Figure 10:**
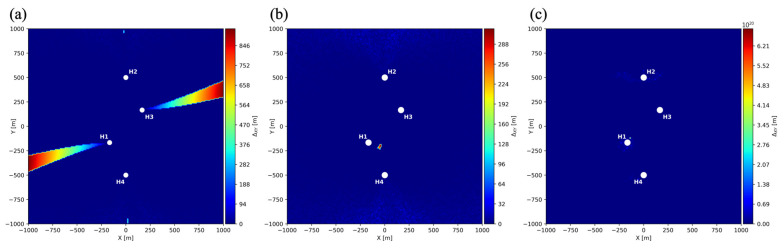
Estimated position error for TDOA values with σ=0.3 m and parallelogram sensor geometry (variant 2).

**Figure 11:**
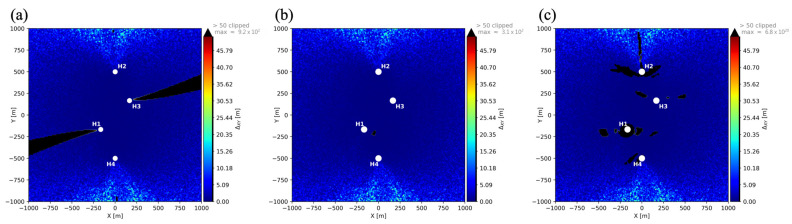
Estimated position error for TDOA values with σ=0.3 m and variant 2 sensor geometry with a max error of 50 m.

Figure 8, Figure 9, Figure 10 and Figure 11 present the estimated position results for TDOA values with σ=0.3 m for two different sensor geometries. Figure 8 and Figure 10 show the full range of error values, while Figure 9 and Figure 11 display the same results but with a compressed scale to allow for better comparison of smaller error values.

**Figure 12:**
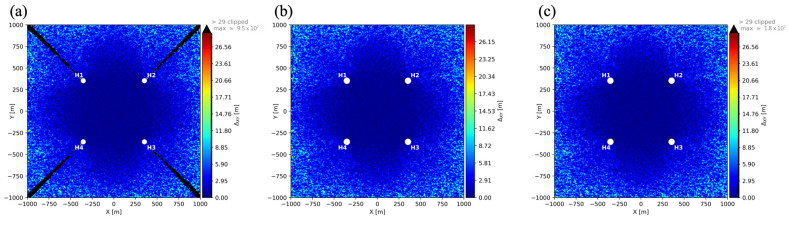
Estimated position error for TDOA values with σ=1 m and variant 1 sensor geometry with a max error of 29 m.

**Figure 13:**
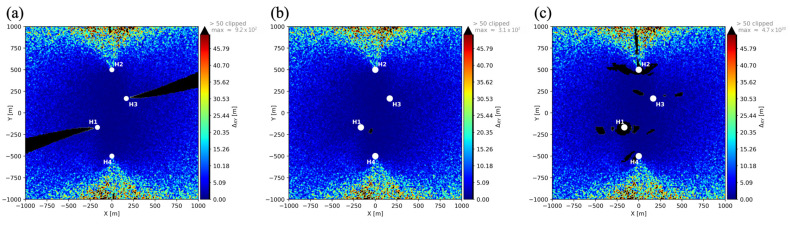
Estimated position error for TDOA values with σ=1 m and variant 2 sensor geometry with a max error of 50 m.

Figure 12 and Figure 13 present the estimated position results for TDOA values with σ=1 m for two different sensor geometries. The figures are shown only with a compressed error scale, as the full-scale versions do not provide additional insight beyond generally higher error values.

**Figure 14:**
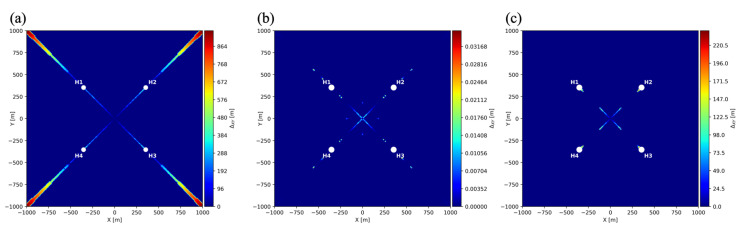
Estimated position error for ideal TDOA and variant 1 sensor geometry.

**Figure 15:**
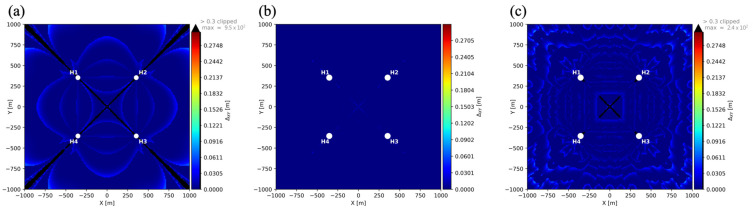
Estimated position error for ideal TDOA and variant 1 sensor geometry with a max error of 0.3 m.

**Figure 16:**
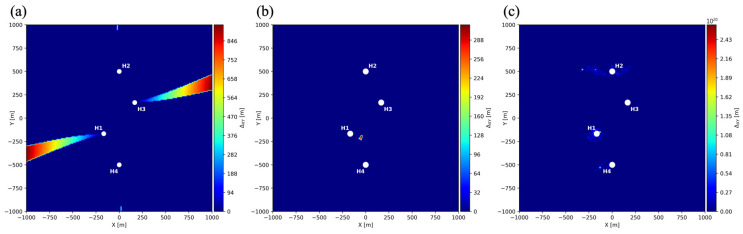
Estimated position error for ideal TDOA and variant 2 sensor geometry.

**Figure 17:**
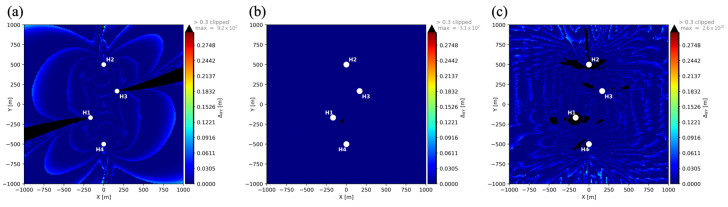
Estimated position error for ideal TDOA and variant 2 sensor geometry with a max error of 0.3 m.

Figure 14, Figure 15, Figure 16 and Figure 17 show the results for ideal (noise-free) TDOA values for both sensor geometries.

For compressed figures, the maximum error scale was set to the maximum error value of the best-performing method, or 50 m in cases where this value exceeded 50 m. An exception applies to the noise-free TDOA results, where the scale limit was set to 0.3 m in order to reveal the spatial distribution of numerical computation errors, which would otherwise appear as a uniform background.

Table 1 presents the basic statistical results for all simulations, while Table 2 shows the mean computation times in Python for each case.

For better visibility, the best-performing values for each geometry variant, σ value, and statistical parameters (Min, Max, Mean, Median, Std, and Error > 50 m) are highlighted in blue while the worst-performing values are in orange for both the 2D and 3D scenarios (Table 1, Table 2, Table 3, Table 4, Table 5, Table 6 and Table 7).

### 5.2. Two-Dimensional Case Discussion

Taking into consideration the results presented in Section 5.1, it can be observed that a large portion of the studied area yields very similar position estimation results regardless of the initial estimate selection method. This is most evident in the figures with a limited maximum error range or, even more clearly, in the results obtained using ideal (noise-free) TDOA values, where in the convergence regions, Method 1 produces nearly zero errors and the other methods yield slightly higher but still very small errors. These are the regions where the NR method consistently converges regardless of the initial estimate.

Conversely, regions shown in black for compressed figures—indicating position estimation error exceeding the applied scale limit—are predominantly the result of NR divergence due to an inappropriate initial estimate, as confirmed by the corresponding full-scale figures where the same regions can exhibit extremely large error values. These problematic areas correspond directly with similar regions in the noisy TDOA results.

The above findings clearly highlight the advantage of Method 1 for initial estimate selection. In the simulated scenarios, it successfully avoided NR divergence in nearly all cases (with only one exception in the second sensor geometry variant). Method 2 also performed well for the square sensor geometry, with only a very small area near the hydrophones showing incorrect results. Unfortunately, for the alternative sensor configuration, Method 2 performed less reliably, and the regions of high estimation error became unpredictable.

In contrast, placing the initial estimate at the center of the sensor formation yielded accurate results within the central area but exhibited a relatively large surrounding region with poor NR convergence.

### 5.3. Three-Dimensional Simulations

All 3D simulations were conducted using six sensors.

In each figure, the applied initial position selection methods are labeled as seen in the 2D case.

Three-dimensional simulations were conducted for the same area and sensor geometries, with the localized object placed at depths of 20 m and 80 m.

**Figure 18:**
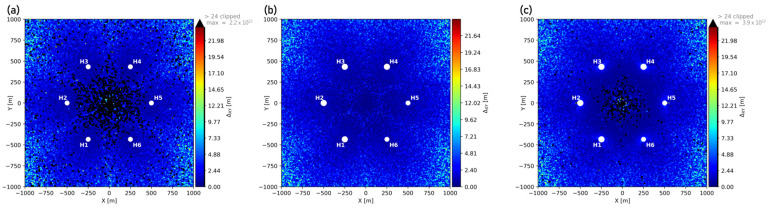
Estimated XY position error for TDOA values with σ=1 m, variant 1 sensor geometry, and localized object at Z=−20 m with a max error of 24 m.

**Figure 19:**
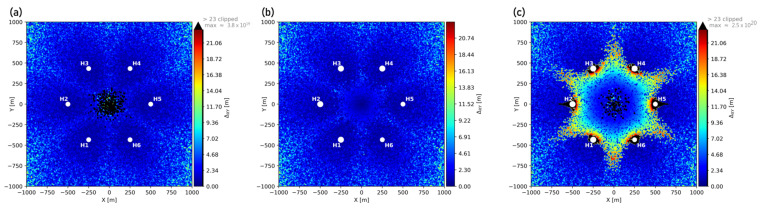
Estimated XY position error for TDOA values with σ=1 m, variant 1 sensor geometry, and localized object at Z=−80 m with a max error of 23 m.

**Figure 20:**
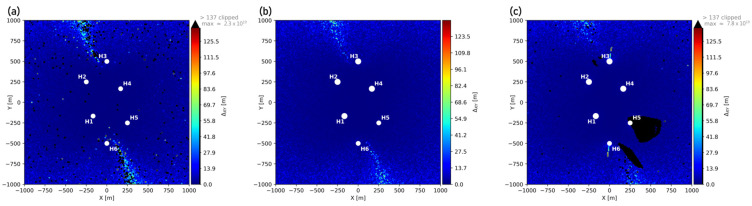
Estimated XY position error for TDOA values with σ=1 m, variant 2 sensor geometry, and localized object at Z=−20 m sensor geometry with a max error of 137 m.

**Figure 21:**
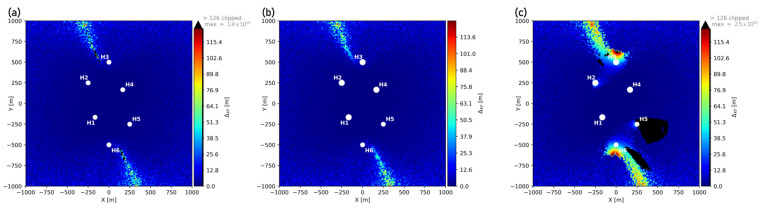
Estimated XY position error for TDOA values with σ=1 m, variant 2 sensor geometry, and localized object at Z=−80 m with a max error of 126 m.

**Figure 22:**
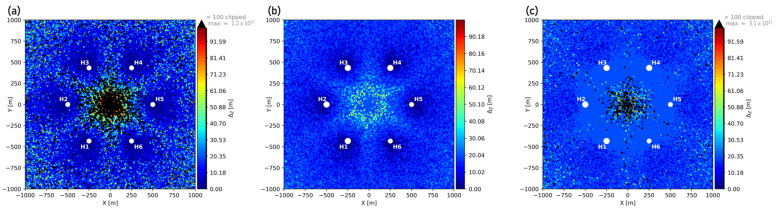
Estimated Z position error for TDOA values with σ=1 m, variant 1 sensor geometry, and localized object at Z=−20 m with a max error of 100 m.

**Figure 23:**
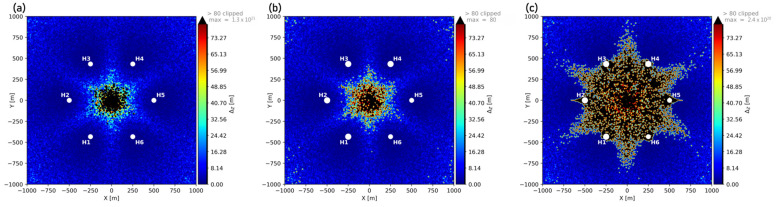
Estimated Z position error for TDOA values with σ=1 m, variant 1 sensor geometry, and localized object at Z=−80 m with a max error of 80 m.

**Figure 24:**
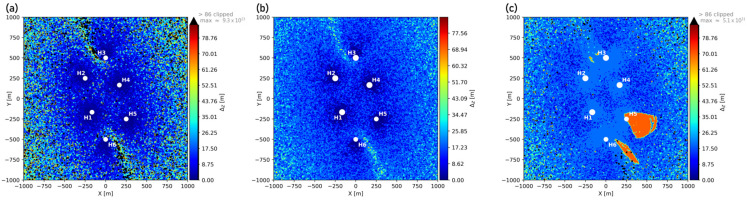
Estimated Z position error for TDOA values with σ=1 m, variant 2 sensor geometry, and localized object at Z=−20 m with a max error of 86 m.

**Figure 25:**
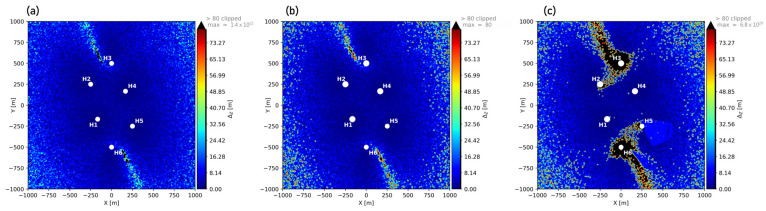
Estimated Z position error for TDOA values with σ=1 m, variant 2 sensor geometry, and localized object at Z=−80 m with a max error of 80 m.

To avoid including an excessive number of figures, only the results with a compressed error scale for σ=1 m are presented, while the results for all tested σ values are provided in the tables. Figure 18, Figure 19, Figure 20 and Figure 21 and Table 3 and Table 4 present the estimated XY position errors, whereas Figure 22, Figure 23, Figure 24 and Figure 25 and Table 5 and Table 6 present the estimated Z position errors.

Additionally, Table 7 shows the mean computation times in Python for each case.

### 5.4. Three-Dimensional Case Discussion

The results presented in Section 5.3 for 3D object position estimation show similar trends to those in Section 5.1 for 2D localization, particularly in the XY plane.

In the 3D XY case, it can again be observed that a large portion of the studied area yields very similar position estimation results regardless of the initial estimate selection method, as indicated by the comparable median error values. However, unlike in the 2D simulations, the areas of improper convergence are concentrated in the center of the variant 1 geometry, whereas they are spread irregularly throughout the analyzed area in the variant 2 geometry. It can be noted that Method 2 exhibits the poorest convergence, while Method 1 performs best. As in the 2D scenario, Method 1 successfully avoids NR divergence in the XY surface of the 3D case. Nevertheless, it is noteworthy that the Guess 0 method tends to produce either very large outliers or very good convergence in the remaining area.

It should also be emphasized that the *Z*-axis results are generally worse. This can be attributed to the hyperboloid geometry and the high sensitivity of TDOA errors at small Z values, which were tested in this study. Still, the general conclusions regarding method comparison remain consistent with those for the XY surface.

An important point not yet mentioned is the higher computational complexity of Method 1, which results in significantly longer processing times, as shown in Table 2 and Table 7. The computations presented in this article were performed without parallelization and on a non-high-performance machine, so faster execution could be expected under optimized conditions. Nevertheless, for real-time applications, this increased computational demand could be critical. For this reason, and based on the test results, one additional method was considered. For the purposes of this article, it is referred to as Method 3, a hybrid approach combining Guess 0 and Method 1, described in the next section.

### 5.5. Method 3

The Method 3 algorithm can be described as follows (Algorithm 3):
**Algorithm 3.** Method 3**Input:** $sensor.coord=\left[\begin{array}{cccc} x_{H_{1}} & x_{H_{2}} & \ldots & x_{H_{N}} \end{array}\right]\;$,     $TDOA=\;\left[\begin{array}{ccccc} TDOA1 & TDOA2 & \ldots & TDOAm & TDOA1 \end{array}\right]$, maximum depth/heightCalculate estimated position using Guess 0 algorithm**if** XY distance from middle of sensor geometry > 100 * geometry radius **or** Z > 2 * max_depth_or_height **do**  Calculate estimated position using Method 1 algorithm**end****Output:** *Final estimated position*

#### 5.5.1. Three-Dimensional Method 3 Simulations

Simulations were conducted for the same area, sensor geometries, and depths as in the previous 3D experiments.

**Figure 26:**
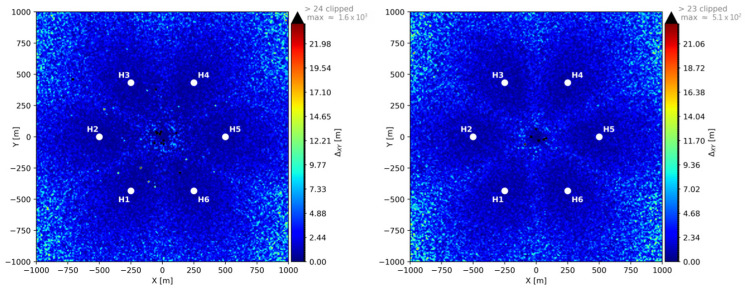
Estimated XY position error for TDOA values with σ=1 m, variant 1 sensor geometry, and localized object at Z=−20 m (**left**) and Z=−80 m (**right**) with a max error of 24 m (**left**) and 23 m (**right**).

**Figure 27:**
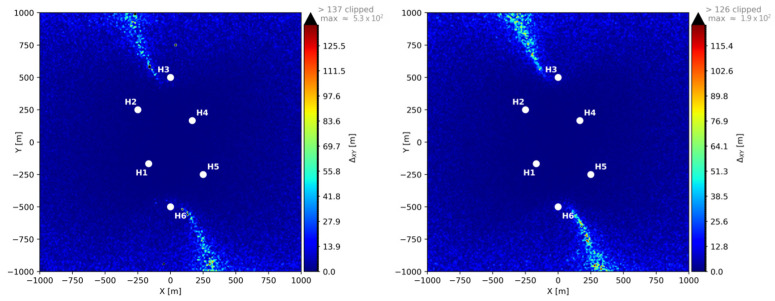
Estimated XY position error for TDOA values with σ=1 m, variant 2 sensor geometry, and localized object at Z=−20 m (**left**) and Z=−80 m (**right**) with a max error of 137 m (**left**) and 126 m (**right**).

**Figure 28:**
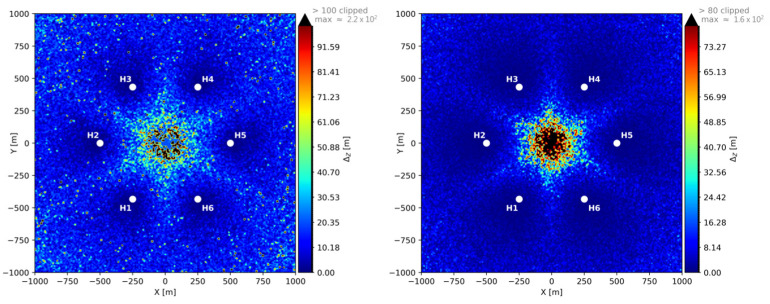
Estimated Z position error for TDOA values with σ=1 m, variant 1 sensor geometry, and localized object at Z=−20 m (**left**) and Z=−80 m (**right**) with a max error of 100 m (**left**) and 80 m (**right**).

**Figure 29:**
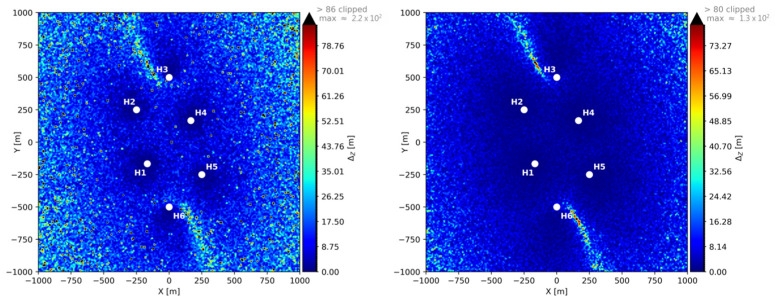
Estimated Z position error for TDOA values with σ=1 m, variant 2 sensor geometry, and localized object at Z=−20 m (**left**) and Z=−80 m (**right**) with a max error of 86 m (**left**) and 80 m (**right**).

To avoid including an excessive number of figures, only results with a compressed error scale for σ=1 m are presented, with the range cut at the same values as seen in Section 5.3 to improve comparability. The results for all tested σ values are summarized in the tables. Figure 26 and Figure 27 and Table 8 present the estimated XY position errors, while Figure 28 and Figure 29 and Table 9 present the estimated Z position errors.

Additionally, Table 10 shows the mean computation times in Python for each case.

#### 5.5.2. Three-Dimensional Method 3 Case Discussion

The results presented in Section 5.5.1 show that Method 3 achieves considerably better performance than Guess 0. However, compared to Method 1, it still produces some individual points with relatively large localization errors. These errors are difficult to eliminate, as they are not easily distinguishable from valid estimates and cannot be classified as clear outliers. This outcome supports the earlier assumptions and suggests that Method 3 may serve as a practical backup approach when computational efficiency is critical since Table 10 demonstrates its notably lower mean computation times.

It should be noted that the computation times presented in Table 10 were obtained in separate simulation runs from those in Table 2 and Table 7, and despite efforts to maintain consistent hardware conditions, minor variations in system load may have influenced the results. Therefore, the absolute values should be treated as approximate, and only relative comparisons within each table are considered reliable. Nevertheless, Table 10 clearly confirms that Method 3 achieves substantially lower computation times than Method 1, while maintaining accuracy close to it.

Based on the overall simulation results, the following practical guidance can be provided for method selection. In practice, Method 1 is recommended when maximum convergence reliability is required and computational resources are not a limiting factor. On the other hand, Method 3 offers a favorable alternative for real-time or resource-constrained applications, providing accuracy close to Method 1 while maintaining substantially lower computation times.

## 6. Conclusions

This study analyzed the performance of different initial estimate selection methods for iterative position estimation algorithm, namely the Newton–Raphson (NR) method, in both 2D and 3D scenarios using TDOA measurements. The results demonstrated that a large portion of the studied domain yields similar and accurate localization regardless of the initial estimate selection method, especially in the XY plane. However, Method 1 consistently achieved the best convergence performance and avoided divergence, while Method 2 showed the weakest results.

For the Z-dimension, position estimation was more challenging due to the geometry of hyperboloids and stronger sensitivity to TDOA errors. Nevertheless, the relative comparison between methods was consistent with the XY plane results. A trade-off between accuracy and computational cost was also highlighted: although Method 1 provided the most robust convergence, it required significantly higher computational time.

To address this issue, Method 3—a hybrid approach combining Guess 0 with Method 1—was proposed. Simulations confirmed that Method 3 offers a favorable compromise: mean position estimation accuracy close to Method 1 while maintaining substantially reduced computation time. Therefore, Method 3 can be considered a suitable candidate for real-time applications where computational efficiency is essential.

## Figures and Tables

**Table 1:** Statistical results for estimated position error [m].

		Geometry Variant 1			Geometry Variant 2		
		σ=0	σ=0.3 m	σ=1 m	σ=0	σ=0.3 m	σ=1 m
**Min**	**Guess 0**	0	0.00248	0.00927	0	0.00176	0.00550
	**Method 1**	0	0.00248	0.00927	0	0.00176	0.00550
	**Method 2**	0	0.00248	0.00927	0	0.00176	0.00550
**Max**	**Guess 0**	947.66774	947.75188	948.01633	924.55959	924.73679	924.79951
	**Method 1**	0.03474	8.12821	28.27857	312.82463	312.24132	313.88119
	**Method 2**	240.41631	178.09561	180.71378	7.0723 × 10^20^	6.7984 × 10^20^	4.72 × 10^20^
**Mean**	**Guess 0**	14.82648	13.23374	13.91974	22.73601	24.73058	29.86613
	**Method 1**	3.9781 × 10^−5^	0.88175	2.93916	0.07540	2.16957	7.04402
	**Method 2**	0.20628	0.94919	3.01030	1.9987 × 10^17^	1.7342 × 10^17^	1.70 × 10^17^
**Median**	**Guess 0**	0.00052	0.56309	1.85126	0.00074	1.07586	3.59883
	**Method 1**	2.8431 × 10^−6^	0.55453	1.82582	4.6344 × 10^−6^	0.99118	3.30565
	**Method 2**	0.00051	0.55557	1.82996	0.00082	1.04776	3.49714
**Std**	**Guess 0**	99.73995	93.29828	87.64742	123.21299	122.74135	123.10107
	**Method 1**	0.00078	0.91031	3.04617	4.59416	5.47371	11.14367
	**Method 2**	5.28459	3.11907	4.28070	4.9864 × 10^18^	3.921 × 10^18^	3.16 × 10^18^
**Error > 50 m**	**Guess 0**	2.53%	1.96%	1.70%	3.68%	3.69%	4.69%
	**Method 1**	0%	0%	0%	0.03%	0.03%	1.03%
	**Method 2**	0.14%	0.05%	0.05%	1.99%	2.00%	3.04%

**Table 2:** Mean Python calculation time [s] for 2D case.

		Geometry Variant 1			Geometry Variant 2		
		σ=0	σ=0.3 m	σ=1 m	σ=0	σ=0.3 m	σ=1 m
**Mean**	**Guess 0**	0.00414	0.00789	0.00833	0.00344	0.00721	0.01060
	**Method 1**	0.06607	0.13498	0.14363	0.08705	0.16853	0.24913
	**Method 2**	0.00489	0.00872	0.00919	0.00468	0.00856	0.01251

**Table 3:** Statistical results for XY estimated position error [m] for localized object at Z=−20 m.

		Geometry Variant 1			Geometry Variant 2		
		σ=0	σ=0.3 m	σ=1 m	σ=0	σ=0.3 m	σ=1 m
**Min**	**Guess 0**	0	0.00069	0.00956	0	0.00232	0.00144
	**Method 1**	0	0.00069	0.00874	0	0.00199	0.00144
	**Method 2**	0	0.00071	0.00452	0	0.00199	0.00470
**Max**	**Guess 0**	2.06 × 10^−9^	6.08 × 10^21^	2.19 × 10^22^	2.63 × 10^18^	1.73 × 10^19^	2.30 × 10^19^
	**Method 1**	0.02405	6.52478	23.64734	5.57 × 10^−10^	37.77783	136.53234
	**Method 2**	44.46808	4.63 × 10^21^	3.87 × 10^22^	5.02 × 10^20^	1.70 × 10^20^	7.83 × 10^19^
**Mean**	**Guess 0**	1.04 × 10^−10^	5.25 × 10^17^	9.73 × 10^17^	8.96 × 10^13^	5.94 × 10^15^	3.42 × 10^16^
	**Method 1**	1.19 × 10^−6^	0.71578	2.20049	4.68 × 10^−12^	1.24138	3.85534
	**Method 2**	0.66963	2.31 × 10^17^	9.83 × 10^17^	2.68 × 10^17^	2.66 × 10^17^	2.61 × 10^17^
**Median**	**Guess 0**	3.96 × 10^−11^	0.51213	1.76089	9.31 × 10^−12^	0.65662	2.22883
	**Method 1**	1.31 × 10^−12^	0.50815	1.48398	9.17 × 10^−13^	0.66716	2.14466
	**Method 2**	0.53141	0.79612	1.57637	0.32069	0.92544	2.34559
**Std**	**Guess 0**	1.90 × 10^−10^	4.68 × 10^19^	1.12 × 10^20^	1.40 × 10^16^	2.23 × 10^17^	5.26 × 10^17^
	**Method 1**	0.00017	0.66414	2.18118	2.20 × 10^−11^	1.74652	5.50762
	**Method 2**	0.77116	3.15 × 10^19^	1.93 × 10^20^	3.14 × 10^18^	2.11 × 10^18^	1.86 × 10^18^
**Error > 50 m**	**Guess 0**	0%	1.06%	3.71%	0.005%	0.24%	1.45%
	**Method 1**	0%	0%	0%	0%	0%	0.21%
	**Method 2**	0%	0.09%	0.73%	2.67%	2.76%	3.01%

**Table 4:** Statistical results for XY estimated position error [m] for localized object at Z=−80 m.

		Geometry Variant 1			Geometry Variant 2		
		σ=0	σ=0.3 m	σ=1 m	σ=0	σ=0.3 m	σ=1 m
**Min**	**Guess 0**	0	0.00098	0.00434	0	0.00243	0.00634
	**Method 1**	0	0.00098	0.00434	0	0.00243	0.00634
	**Method 2**	0	0.00413	0.00434	0	0.00271	0.00634
**Max**	**Guess 0**	6.28 × 10^−10^	2.43 × 10^20^	3.83 × 10^19^	9.11 × 10^18^	1.15 × 10^19^	1.42 × 10^19^
	**Method 1**	0.37792	7.37263	22.09291	3.43 × 10^−8^	85.80746	125.03751
	**Method 2**	112.77752	1.11 × 10^19^	2.52 × 10^20^	5.29 × 10^19^	8.02 × 10^19^	2.53 × 10^20^
**Mean**	**Guess 0**	5.97 × 10^−11^	1.09 × 10^16^	1.20 × 10^16^	9.34 × 10^14^	6.78 × 10^14^	2.53 × 10^15^
	**Method 1**	1.87 × 10^−5^	0.72729	2.39486	3.31 × 10^−11^	1.33196	4.49981
	**Method 2**	2.40521	3.54 × 10^14^	1.12 × 10^16^	2.43 × 10^17^	2.43 × 10^17^	2.58 × 10^17^
**Median**	**Guess 0**	2.58 × 10^−11^	0.52080	1.72674	5.96 × 10^−12^	0.66254	2.23499
	**Method 1**	9.70 × 10^−12^	0.52151	1.71704	2.77 × 10^−12^	0.66254	2.24448
	**Method 2**	1.71 × 10^−11^	0.79609	2.49356	5.67 × 10^−12^	0.76638	2.61461
**Std**	**Guess 0**	8.64 × 10^−11^	1.4 × 10^18^	3.51 × 10^17^	8.55 × 10^16^	7.49 × 10^16^	1.48 × 10^17^
	**Method 1**	0.00266	0.66725	2.21666	3.87 × 10^−10^	2.44396	7.68037
	**Method 2**	6.65049	5.6 × 10^16^	1.29 × 10^18^	1.78 × 10^18^	1.76 × 10^18^	2.36 × 10^18^
**Error > 50 m**	**Guess 0**	0%	0.23%	0.74%	0.02%	0.03%	0.50%
	**Method 1**	0%	0%	0%	0%	0.004%	0.62%
	**Method 2**	0.31%	0.45%	0.61%	5.38%	5.58%	5.90%

**Table 5:** Statistical results for Z estimated position error [m] for localized object at Z=−20 m.

		Geometry Variant 1			Geometry Variant 2		
		σ=0	σ=0.3 m	σ=1 m	σ=0	σ=0.3 m	σ=1 m
**Min**	**Guess 0**	0.00122	0.01401	0.00006	0.00101	0.00002	0.00015
	**Method 1**	0.00004	0.01426	0.00148	0.00031	0.00011	0.00026
	**Method 2**	0.00033	0.05134	0.00305	20	0.00002	0.00122
**Max**	**Guess 0**	40	6.08 × 10^21^	1.22 × 10^22^	2.18 × 10^20^	2.83 × 10^21^	9.32 × 10^23^
	**Method 1**	70	99.67393	99.91009	0.00047	38.48865	85.48991
	**Method 2**	70	4.63 × 10^21^	3.11 × 10^21^	4.70 × 10^31^	4.21 × 10^34^	5.07 × 10^31^
**Mean**	**Guess 0**	0.00370	8.44 × 10^17^	2.84 × 10^18^	5.43 × 10^15^	1.68 × 10^17^	2.41 × 10^19^
	**Method 1**	0.00313	7.84931	14.40820	0.00042	8.56505	15.29117
	**Method 2**	19.97204	2.51 × 10^17^	3.94 × 10^17^	1.81 × 10^27^	1.04 × 10^30^	1.70 × 10^27^
**Median**	**Guess 0**	0.00276	3.40441	9.90611	0.00277	3.82901	11.47741
	**Method 1**	0.00042	4.16985	18.93162	0.00042	5.16913	20
	**Method 2**	20	20.00891	20	20	20	20
**Std**	**Guess 0**	0.19899	4.91 × 10^19^	1.01 × 10^20^	1.09 × 10^18^	1.58 × 10^19^	4.64 × 10^21^
	**Method 1**	0.37560	8.26913	9.27873	5.60 × 10^−6^	7.81168	9.14668
	**Method 2**	0.80307	3.17 × 10^19^	2.12 × 10^19^	2.64 × 10^29^	2.09 × 10^32^	2.63 × 10^29^
**Error > 50 m**	**Guess 0**	0%	2.50%	10.11%	0.005%	2.22%	10.05%
	**Method 1**	0.002%	0.25%	0.61%	0%	0%	0.51%
	**Method 2**	0.002%	0.19%	1.77%	2.61%	2.84%	4.73%

**Table 6:** Statistical results for Z estimated position error [m] for localized object at Z=−80 m.

		Geometry Variant 1			Geometry Variant 2		
		σ=0	σ=0.3 m	σ=1 m	σ=0	σ=0.3 m	σ=1 m
**Min**	**Guess 0**	0.00001	8.96 × 10^−6^	0.00007	4.14 × 10^−6^	0.00012	0.00012
	**Method 1**	0.00019	0.00002	0.00006	0.00010	9.78 × 10^−6^	7.09 × 10^−6^
	**Method 2**	0.00033	0.00001	0.00007	0.00033	7.63 × 10^−5^	7.92 × 10^−6^
**Max**	**Guess 0**	20	5.57 × 10^20^	1.29 × 10^21^	2.09 × 10^19^	8.13 × 10^17^	1.37 × 10^22^
	**Method 1**	80	80	80	0.00146	80	80
	**Method 2**	80	5.53 × 10^19^	2.38 × 10^20^	1.43 × 10^31^	1.24 × 10^29^	6.84 × 10^29^
**Mean**	**Guess 0**	0.00107	2.04 × 10^16^	7.68 × 10^16^	7.2191 × 10^14^	4.23237 × 10^13^	3.53 × 10^17^
	**Method 1**	0.00456	2.43369	6.83141	0.00035	1.74777	7.47651
	**Method 2**	17.7467	1.86 × 10^15^	2.50 × 10^16^	5.47 × 10^26^	3.07 × 10^24^	1.69 × 10^25^
**Median**	**Guess 0**	0.00057	0.88398	2.92998	0.00057	1.03444	3.52372
	**Method 1**	0.00035	0.88471	2.94085	0.00035	1.03431	3.56546
	**Method 2**	0.00057	1.14153	3.86475	0.00057	1.21998	4.28309
**Std**	**Guess 0**	0.09949	2.83 × 10^18^	6.70 × 10^18^	1.08 × 10^17^	4.87187 × 10^15^	6.84 × 10^19^
	**Method 1**	0.56505	8.75938	14.56129	2.02 × 10^−5^	2.69965	13.47539
	**Method 2**	33.23768	2.85 × 10^17^	1.64 × 10^18^	7.49 × 10^28^	6.17 × 10^26^	3.40 × 10^27^
**Error > 50 m**	**Guess 0**	0%	0.60%	1.87%	0.02%	0.03%	0.30%
	**Method 1**	0.005%	1.17%	3.45%	0%	0.04%	2.55%
	**Method 2**	22.18%	21.13%	20.91%	6.22%	6.45%	9.11%

**Table 7:** Mean Python calculation time [s] for 3D case.

		Geometry Variant 1			Geometry Variant 2		
		σ=0	σ=0.3 m	σ=1 m	σ=0	σ=0.3 m	σ=1 m
**Mean**	**Guess 0**	0.10929	0.10044	0.09759	0.12181	0.09494	0.09552
	**Method 1**	0.67805	0.63696	0.62699	0.73782	0.59359	0.60178
	**Method 2**	0.07542	0.07724	0.07225	0.06593	0.06727	0.07084

**Table 8:** Statistical results for XY estimated position error [m].

		Geometry Variant 1			Geometry Variant 2		
		σ=0	σ=0.3 m	σ=1 m	σ=0	σ=0.3 m	σ=1 m
**Min**	**Z = −20 m**	0	0.00271	0.00501	0	0.00145	0.00636
	**Z = −80 m**	0	0.00271	0.00501	0	0.00145	0.00636
**Max**	**Z = −20 m**	2.06 × 10^−9^	662.04500	1590.92337	4.01 × 10^−7^	492.52422	530.21164
	**Z = −80 m**	6.28 × 10^−10^	603.57105	508.04236	0.14455	87.53714	192.00429
**Mean**	**Z = −20 m**	1.04 × 10^−10^	0.77967	2.47963	1.68 × 10^−10^	1.27585	4.12479
	**Z = −80 m**	5.97 × 10^−11^	0.82708	2.44076	7.17 × 10^−6^	1.31693	4.36278
**Median**	**Z = −20 m**	3.96 × 10^−11^	0.49378	1.56689	9.31 × 10^−12^	0.65559	2.16244
	**Z = −80 m**	2.58 × 10^−11^	0.51798	1.71114	5.96 × 10^−12^	0.66259	2.20160
**Std**	**Z = −20 m**	1.90 × 10^−10^	6.15276	13.47963	2.93 × 10^−9^	3.96753	8.20399
	**Z = −80 m**	8.64 × 10^−11^	7.20446	5.70173	0.00102	2.28643	7.17854
**Error > 50 m**	**Z = −20 m**	0%	0.02%	0.03%	0%	0.005%	0.29%
	**Z = −80 m**	0%	0.01%	0.02%	0%	0.01%	0.42%

**Table 9:** Statistical results for Z estimated position error [m].

		Geometry Variant 1			Geometry Variant 2		
		σ=0	σ=0.3 m	σ=1 m	σ=0	σ=0.3 m	σ=1 m
**Min**	**Z = −20 m**	0.00122	4.49 × 10^−6^	0.00047	0.00042	2.19 × 10^−6^	0.00100
	**Z = −80 m**	1.21 × 10^−5^	1.06 × 10^−5^	6.28 × 10^−5^	4.14 × 10^−6^	9.91 × 10^−5^	1.98 × 10^−5^
**Max**	**Z = −20 m**	40	212.40457	219.72746	0.01482	215.58773	218.81609
	**Z = −80 m**	20	159.51806	158.61869	4.66814	80	132.64629
**Mean**	**Z = −20 m**	0.00371	5.72131	13.24489	0.00275	6.37217	14.75289
	**Z = −80 m**	0.00107	2.09685	5.89353	0.00080	1.72724	5.83897
**Median**	**Z = −20 m**	0.00276	3.22893	9.73799	0.00277	3.78333	10.94341
	**Z = −80 m**	0.00057	0.87914	2.91941	0.00057	1.04253	3.48992
**Std**	**Z = −20 m**	0.19899	9.72578	16.89711	0.00033	9.38043	18.21775
	**Z = −80 m**	0.09949	6.82211	11.67596	0.03285	2.29851	7.26794
**Error > 50 m**	**Z = −20 m**	0%	0.59%	2.55%	0%	0.54%	2.72%
	**Z = −80 m**	0%	0.62%	1.78%	0%	0.02%	0.32%

**Table 10:** Mean Python calculation time [s] for Method 3.

		Geometry Variant 1			Geometry Variant 2		
		σ=0	σ=0.3 m	σ=1 m	σ=0	σ=0.3 m	σ=1 m
**Mean**	**Method 3**	0.10724	0.08506	0.05420	0.04437	0.04762	0.05375

## Data Availability

Data are contained within the article.

## Abbreviations

The following abbreviations are used in this manuscript:

CCF	Cross-Correlation Function
LTE	Long Term Evolution
NR	Newton–Raphson
RF	Radio Frequency
TDOA	Time Difference of Arrival
